# Cardiodynamic Interactions between Two S1P_1_ Receptor Modulators in an Experimental Clinical Setting: Different Pharmacokinetic Properties as an Opportunity to Mitigate First-Dose Heart Rate Effects

**DOI:** 10.3390/ijms20133232

**Published:** 2019-07-01

**Authors:** Pierre-Eric Juif, Mike Ufer, Jasper Dingemanse

**Affiliations:** Department of Clinical Pharmacology, Idorsia Pharmaceuticals Ltd, Allschwil CH-4123, Switzerland

**Keywords:** S1P_1_ receptor modulators, first-dose effect, pharmacokinetics

## Abstract

A decrease in heart rate (HR) is a well-established first-dose effect of sphingosine-1-phosphate subtype 1 receptor (S1P1R) modulators. For compounds with a short half-life (*t*_1/2_), this can be mitigated by gradual up-titration to therapeutic doses, whereas this is not required for compounds with a long *t*_1/2_ due to the less pronounced first-dose-related negative chronotropic effects. Based on this conceptual framework, this mechanistic study investigated whether first-dose HR effects of ponesimod (*t*_1/2_ ~32 h) can be mitigated by prior administration of cenerimod (*t*_1/2_ ~415 h). Healthy subjects (*n* = 12) were randomly assigned to active or placebo (2:1 ratio). Active treatment consisted of a single dose of 10 mg ponesimod on Day 1, 18, and 37 and multiple-dose administration of 2 mg once daily cenerimod (Day 9–36). Placebos of cenerimod and ponesimod were used as reference treatment. Cardiodynamic parameters were derived from 24 h Holter electrocardiogram (ECG) assessments on Day 1, 9, 10, 18, 36, and 37. Ponesimod (10 mg) alone triggered a transient mean decrease from baseline in hourly mean HR of 17 bpm. In contrast, decreases of 5.0 and 4.8 bpm were observed when ponesimod was given at near half steady-state (Day 18) or steady-state (Day 37) cenerimod, respectively. Hourly mean HR decreased after first administration of cenerimod and placebo was 7.4 and 4.0 bpm, respectively. Treatment with ponesimod and cenerimod alone or in combination was safe and tolerated. First-dose-related negative chronotropic effects of ponesimod were less pronounced when administered after initiation of cenerimod suggesting mitigation of this class-related liability.

## 1. Introduction

Sphingosine-1-phosphate subtype 1 receptor (S1P1R) modulators have shown efficacy in the treatment of autoimmune diseases, e.g., multiple sclerosis, inflammatory bowel disease, or chronic plaque psoriasis [1]. Chronic treatment with these modulators triggers sustained internalization of this receptor and induces a long-lasting inhibition of lymphocyte egress from lymphoid organs [2].

After first-dose administration, several class effects on the cardiovascular system have been reported. First-dose-related reduction of heart rate (HR) and blood pressure (BP) are well-established effects that may potentially lead to adverse events (AEs) such as bradycardia, atrioventricular (AV) blocks, and hypotension [1]. Mechanistically, this is due to the activation of the S1P1R leading to the activation of the G-protein coupled inwardly rectifying potassium channels (GIRK) that regulate pacemaker activity resulting in a negative chronotropic effect and delayed AV conduction [3,4].

First-dose-related reductions in HR are applicable to S1P1R modulators exhibiting short half-lives (*t*_1/2_), e.g., ponesimod [5] or siponimod [6], and can be mitigated using an up-titration regimen. In contrast, S1P1R modulators with a long *t*_1/2_, e.g., cenerimod and fingolimod, are less prone to such first-dose effects, presumably due to the so-called “built-in up-titration” [1,7].

Mechanistically, another approach to potentially attenuate first-dose-related negative chronotropic effects would, therefore, be to administer a short *t*_1/2_ S1P1R modulator after receptor internalization and desensitization elicited by a long *t*_1/2_ compound. To investigate this approach, cenerimod (i.e., long *t*_1/2_ S1P1R modulator) and ponesimod (i.e., short *t*_1/2_ S1P1R modulator) were administered to healthy subjects. Cenerimod is being investigated in a dose-finding study in systemic lupus erythematosus patients (NCT03742037), while ponesimod has been investigated in chronic plaque psoriasis [8] and in multiple sclerosis patients [9].

This conceptual approach was investigated in this study using a parallel-group design. Heart rate and other safety parameters were evaluated for a single oral dose of ponesimod administered alone (Day 1), at near half steady-state (Day 18), and at steady-state exposure to cenerimod (Day 37).

## 2. Results

### 2.1. Demographics

All subjects were Caucasian except for one Asian subject in the placebo group. Demographic variables were similar between placebo- and active-treated subjects. The mean (SD) age and BMI were 25 (3.8) years and 26.1 (3.3) kg/m^2^, respectively.

### 2.2. Cardiodynamics

#### 2.2.1. Hourly Mean HR

When ponesimod was administered alone, the pre-dose hourly mean (SD) HR on Day 1 was 74.0 (5.8) bpm. The nadir in hourly mean (SD) HR observed 2 h after single-dose administration of ponesimod was 57.0 (4.7) bpm.

**Figure 1:**
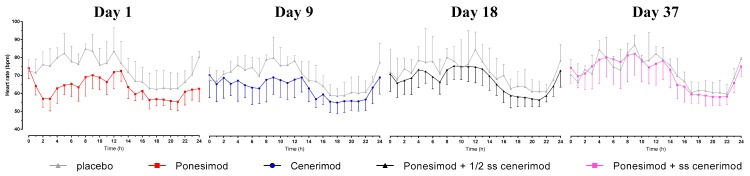
Hourly mean heart rate profile over 24 h by treatment group. Arithmetic mean heart rate versus time profiles following single-dose administration of 10 mg ponesimod alone (Day 1, red curve), at approximately 50% steady state of 2 mg once daily cenerimod (Day 18, black curve), at steady-state cenerimod (Day 37, pink curve), and following first-dose administration of cenerimod (Day 9, blue curve), *n* = 8. Placebo data of the corresponding days are displayed in grey (*n* = 4). Data were derived from 24 h Holter ECGs. Error bars represent standard deviation.

When ponesimod was administered at approximately 50% of steady-state (Day 18, i.e., 9 days after first administration of cenerimod) and at steady-state concentration of cenerimod (Day 37, i.e., one day after last administration of cenerimod), the hourly mean (SD) HR decreased from 71.1 (9.7) to 66.1 (8.0) bpm and from 74.6 (8.6) bpm to 69.8 (6.4) bpm, respectively (Figure 1).

Prior to the first administration of cenerimod on Day 9, the hourly mean (SD) HR was 70.4 (12.6) bpm. It decreased post-dose with a nadir of 63.0 (8.5) bpm observed at 7 h post-dose.

In the placebo group, the pre-dose hourly mean (SD) HR on Day 1 and 9 was 72.5 (6.2) bpm and 67.7 (6.8) bpm, respectively, and hence, similar to the pre-dose hourly mean HR in the active group. There was essentially no change post administration of placebo indicated by a nadir of 71.5 (7.6) bpm and 67.0 (6.0) bpm on Day 1 and 9, respectively.

This was also the case on Day 18 and 37 indicated by post-dose decreases of 72.3 (12.5) to 68.0 (12.0) bpm and 70.7 (8.5) to 66.7 (10.2) bpm, respectively. On these days, the magnitude of these decreases was comparable to that observed with active treatment.

#### 2.2.2. HR_AUEC_

The mean (SD) HR_AUEC_ was lower when ponesimod was administered alone on Day 1 (781.0 (73.2) bpm·h) compared to administration of ponesimod at approximately 50% of steady-state (857 (77) bpm·h, *p* > 0.05 Day 1 versus Day 18) and at steady-state concentration of cenerimod (926 (131) bpm·h, *p* < 0.05 Day 1 versus Day 37). Following the first administration of cenerimod, the mean (SD) HR_AUEC_ was 798 (106) bpm·h (*p* > 0.05 Day 1 versus Day 9).

**Figure 2:**
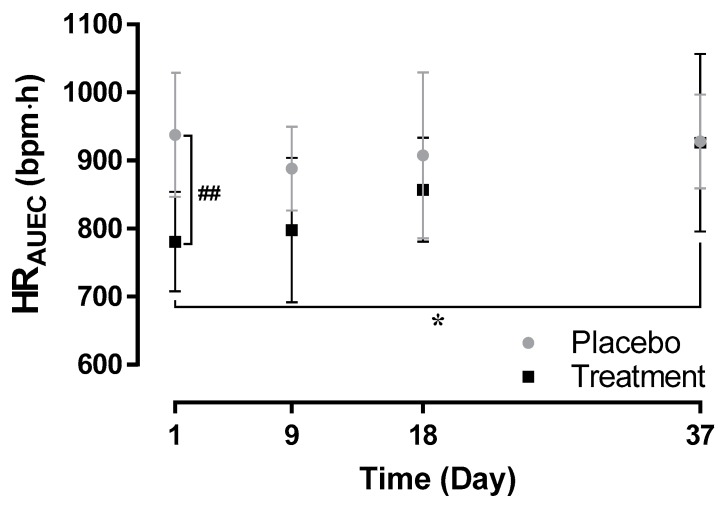
Hourly mean heart rate area under the effect curve (HR_AUEC_) based on the 0–12 h interval by treatment group. Arithmetic mean HR_AUEC_ from 0–12 h following single-dose administration of 10 mg ponesimod alone (Day 1), at approximately 50% steady state of 2 mg once daily cenerimod (Day 18), at steady-state cenerimod (Day 37), and following first-dose administration of cenerimod (Day 9). Active treatment is displayed in black, *n* = 8. Placebo data of the corresponding days are displayed in grey, *n* = 4. Data were derived from 24 h Holter ECGs. Error bars represent standard deviation. * *p* < 0.05 Treatment Day 1 versus Day 37; ^##^ *p* < 0.01 placebo versus treatment on Day 1; Student’s *t*-test.

Following administration of placebo, the mean (SD) HR_AUEC_ ranged between 888 (62) bpm·h and 938 (92) bpm·h (Figure 2). When compared to placebo, HR_AUEC_ was significantly lower following active treatment only after the first administration of ponesimod on Day 1 (*p* < 0.01) but not on Day 9, 18, and 37.

These cardiodynamic data indicate that first-dose cardiodynamic effects of ponesimod were mitigated following initiation of cenerimod.

### 2.3. Pharmacokinetic Assessments

**Figure 3:**
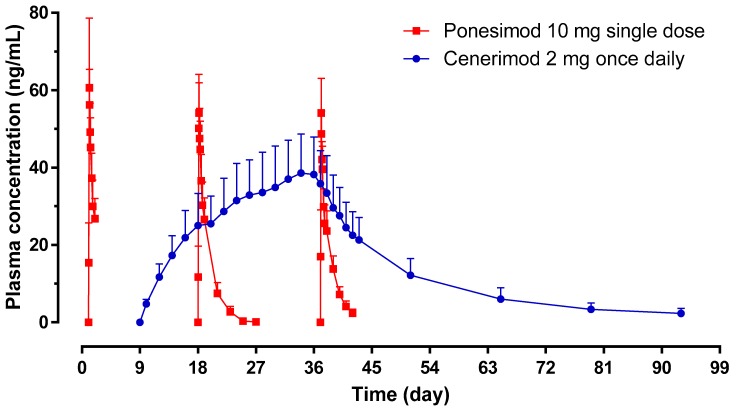
Plasma concentration–time profiles of cenerimod and ponesimod. Arithmetic mean plasma concentration–time profiles of ponesimod (red curves) and trough concentration of cenerimod (blue curve) (*n* = 8). Error bars represent standard deviations.

The pharmacokinetic (PK) profiles of ponesimod on Day 1, 18, and 37 showed similar exposure as reflected by the geometric mean ratio (Day 18/Day 1 and Day 37/Day 1) of AUC_0__–24_ and C_max_ (Table 1; Figure 3). The median *t*_max_ was comparable between Day 1 (3.25 h), Day 18 (4.00 h), and Day 37 (2.50 h). The geometric mean (95% CI) *t*_1/2_ of ponesimod was similar on Day 18 (29.0 (25.1–33.5) h) and Day 37 (28.2 (24.7–32.2) h).

Exposure to cenerimod (AUC_0__–24_ and C_max_) showed accumulation of 5 to 7 fold between Day 9 and Day 36. The geometric mean (95% CI) *t*_1/2_ measured on Day 37 (i.e., day after last administration of cenerimod) was 404 (340–481) h (Table 1).

These PK data indicate that there were no PK interactions between cenerimod and ponesimod.

### 2.4. Lymphocyte Count Assessments

In the active group, the mean (SD) total lymphocyte count at baseline was 1.94 (0.37) × 10^9^ cells/L. On Day 1, the time to maximum effect was 4 h after dosing with ponesimod and the mean (SD) total lymphocyte count was 1.23 (0.35) × 10^9^ cells/L. From Day 9 to Day 36, the total lymphocyte count decreased from 1.99 (0.51) to 0.66 (0.25) × 10^9^ cells/L. A complete recovery of lymphocyte count was observed at End-of-Study (EOS, i.e., within 40 days after last administration of cenerimod).

**Figure 4:**
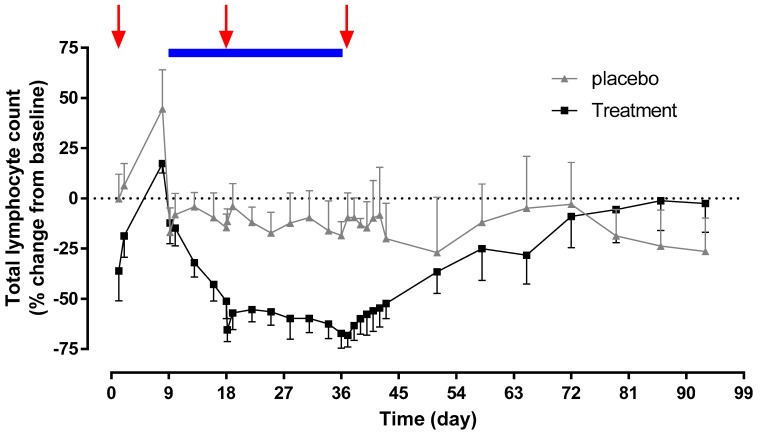
Total lymphocyte count–time profile. Arithmetic mean percentage change from baseline in total lymphocyte count versus time profiles after active treatment (black curve, *n* = 8) or placebo (grey curve, *n* = 4). Single-dose administration of ponesimod is indicated by red arrows and the duration of multiple-dose administration of cenerimod is indicated by the horizontal blue bar. Error bars represent standard deviations.

In the placebo group, the total lymphocyte count did not change to a large extent during the course of the study (Figure 4).

Lymphocyte counts decreased following administration of cenerimod or ponesimod alone. A marginally greater decrease in lymphocyte count was observed when ponesimod was administered on top of cenerimod.

### 2.5. Safety Assessments

Out of the 12 enrolled subjects (4 on placebo, 8 on active), a total of 10 subjects (4 on placebo, 6 on active) reported at least one AE. The most common AEs were headache (2 subjects on placebo, 4 subjects on active) and dizziness (1 subject on placebo, 4 subjects on active). 

Most AEs were of mild intensity (3 of moderate intensity, no severe AEs) and considered related to treatment. Each AE was fully resolved at EOS except for a case of nasopharyngitis that was still ongoing.

One subject discontinued the study due to the presence of AEs (mild abdominal discomfort, diarrhea, and nausea). This occurred on Day 7 in a subject treated with placebo. There were no AEs of special interest during the course of the study. 

Except for the established effects of S1P1R modulators (i.e., lymphocyte count reduction, HR changes), no clinically relevant changes in clinical laboratory parameters or vital sign data including BP were reported.

Ponesimod and cenerimod administered alone and in combination were safe and well tolerated.

## 3. Discussion

This conceptual study aimed at investigating whether priming the S1P1R with the long *t*_1/2_ compound cenerimod may mitigate first-dose HR effects of the short t_1/2_ compound ponesimod. Heart rate effects of ponesimod (10 mg single dose) were compared when given alone versus given after 10 and 27 days of dosing with cenerimod (2 mg o.d.). The dose of cenerimod was selected since it was the dose in the Phase 2 studies (NCT02472795 and NCT03742037) based on its PK/PD profile [7]. The dose of 10 mg ponesimod was selected since it was used as a maintenance dose in earlier Phase 2 trials and, hence, was considered to be potentially therapeutically relevant [7,10,11]. For the pivotal trials, ponesimod was initiated at a dose of 2 mg and the maintenance dose of 20 mg ponesimod was investigated (NCT02425644 and NCT02907177). At these doses, both compounds given alone or in combination were safe and tolerated based on AEs (including absence of AEs of special interest) and other safety data.

The main finding of the present study is that HR was reduced by approximately 17 bpm following administration of ponesimod alone, whereas a HR reduction of only approximately 5 bpm was observed when ponesimod was administered at half-steady-state or steady-state exposure to cenerimod. The first dose of cenerimod alone led to a decrease of approximately 7 bpm. The cardiodynamic profile as reflected by the time to nadir of 2–3 h for ponesimod and 6–7 h for cenerimod is in accordance with the plasma concentration-time profile, i.e., a *t*_max_ of 2.5–4 h for ponesimod and 2.5–6 h for cenerimod.

Disappearance of HR reduction is attributed to the development of tolerance due to the receptor internalization and in turn desensitization of the S1P receptor system following repeated dosing [7,12,13]. In this study, repeated administration of cenerimod led to a mean maximum decrease in HR of 1.5 bpm (Day 36), which was similar to the decrease observed following placebo. In addition, the first-dose effect of ponesimod disappeared after 10 and 27 days of cenerimod o.d. administration. 

As shown in Figure 3 and based on a previous study [7], steady-state conditions of cenerimod were reached on Day 36 and approximately 50% of steady-state conditions were reached on Day 18. These time points were selected to assess whether steady-state exposure to cenerimod would be required to appropriately mitigate first-dose HR effects of ponesimod. Since the extent of HR reduction on Day 18 and Day 37 was similar, steady-state exposure to cenerimod appears not to be required for mitigation of first-dose HR effects of ponesimod. 

The validity of this study is indicated by the fact that the PK parameters of cenerimod and ponesimod were both in line with those of previous studies [7,10]. Multiple-dose administration of cenerimod did not affect the PK of ponesimod as indicated by similar PK parameters obtained when given alone (Day 1) or upon concomitant cenerimod exposure. Therefore, the cardiodynamic results of this study were not confounded by any PK interaction between the two S1P1R modulators. Moreover, the extent of lymphocyte count reduction was also in line with data from previous studies [7,10]. Following attainment of steady-state exposure to cenerimod in the present study, the total lymphocyte count returned to baseline values within 8 to 40 days after last study treatment administration of ponesimod and cenerimod, respectively, which has previously been observed with these S1P1R modulators [1,7,10,14]. In addition, the magnitude of decrease in HR triggered by ponesimod and cenerimod and the time to nadir are in line with previously published data [1,7,14].

The utility of the investigated approach to mitigate first-dose effects may be challenged from a clinical practice perspective. In the present study, the combination of cenerimod followed by ponesimod led to decreases in HR of approximately 7 and 5 bpm after first administration of cenerimod and ponesimod, respectively. In a study investigating the first-dose effect of two up-titration regimens, the use of a newly developed up-titration regimen (incremental dose increase from 2 to 20 mg in nine steps) [5] led to a mean decrease of 6 bpm after initiation with 2 mg ponesimod. The present study investigated whether ponesimod-related first-dose effects can be mitigated also by another S1P1R modulator cenerimod. Hence, the extent of first-dose changes in HR triggered by ponesimod appears similar when given after up-titration or upon concomitant exposure to cenerimod. The present study provides insights in the case of switching from one S1P1R modulator to another one, since these first-dose negative chronotropic effects are class effects and may not be of concern under such scenarios.

In conclusion, the first-dose related chronotropic effects of S1P1R modulators may be of clinical concern and have to be carefully monitored in all patients for the marketed S1P modulator fingolimod [15]. In the case of siponimod, which has recently been approved for the treatment of active secondary progressive multiple sclerosis, extensive first-dose monitoring is not required unless patients have certain pre-existing cardiac conditions (sinus bradycardia, first- or second-degree (Mobitz type I) AV block, or a history of myocardial infarction or heart failure) [16]. In order to improve the safety of S1P1R modulators, up-titration regimens have been developed for those S1P1R modulators with a short *t*_1/2_ to mitigate first-dose HR effects. Such regimens are now well established for ponesimod and siponimod, both characterized by a short *t*_1/2_ [5,6,12,14,17,18]. As revealed in this study, prior exposure to cenerimod effectively mitigated first-dose HR effects of the short *t*_1/2_ S1P1R modulator ponesimod. The study results suggest that the mechanism of desensitization and development of tolerance is class-related and not compound-related suggesting that the concept is translatable to other S1P1R modulators, i.e., desensitization by any S1P1R modulator may lead to mitigation of first-dose effects elicited by another S1P1R modulator. This concept should be considered in case a switch in treatment with an S1P1R modulator is clinically indicated.

## 4. Methods

### 4.1. Subjects

Healthy male and female subjects (non-pregnant, non-lactating) aged between 18 and 50 years with a body mass index between 18.0 and 30.0 kg/m^2^ were enrolled in this study. The screening visit included recording of the medical and drug use history, physical examination, assessments of body weight and height, clinical laboratory, vital sign, and standard electrocardiogram (ECG) data. 

At screening, subjects had to have PR interval ≤ 200 ms, HR 55–90 bpm, systolic (SBP) and diastolic (DBP) BP 100–150 and 50–90 mmHg, respectively, and a normal total lymphocyte count (>1.0 × 10^9^ lymphocytes/L).

Written informed consent was obtained from each subject prior to any study procedure. The protocol was approved by the Plymouth Independent Ethics Committee (UK) (date of initial protocol from Institutional Review Board (IRB): 24 February 2014). This study was performed according to Good Clinical Practice and in accordance with the principles of the Declaration of Helsinki.

### 4.2. Study Design

This single-center, randomized, double-blind, placebo-controlled study had a parallel-group design. A total of 12 healthy subjects participated in the study and were assigned to active treatment (i.e., ponesimod alone and in combination with cenerimod) or placebo according to a 2:1 ratio. A total of 4 females and 8 males participated in the study: 2 females and 2 males received placebo and 2 females and 6 males received active treatment.

**Figure 5:**
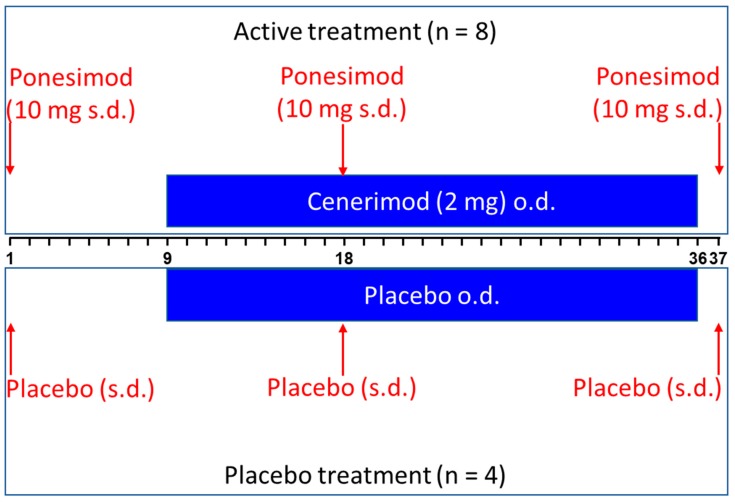
Study design. The red arrows represent the time of administration of ponesimod or its placebo (i.e., Day 1, 18, and 37). The blue bar represents the duration of o.d. administration of cenerimod or its placebo (from Day 9 to Day 36). o.d. = once daily; s.d. = single dose.

Active treatment consisted of a single, oral dose of 10 mg ponesimod on Day 1, 18, and 37 and multiple, oral dose administration of 2 mg once daily (o.d.) cenerimod (Day 9–36, Figure 5). Placebos of cenerimod and ponesimod were administered at the corresponding study days.

Subjects remained in the clinic from Day 1 to Day 2 and from Day 8 until Day 43. They were discharged if this was allowed on the basis of their medical condition. Subjects returned to the clinic for outpatient visits on Days 51, 58, 65, 72, 79, 86, and 93 (End-of-Study (EOS) visit).

### 4.3. Cardiodynamic Assessments

Cardiodynamic parameters (hourly mean HR and area under the effect curve (HR_AUEC_)) were obtained from 24 h Holter ECG assessments (Days 1, 9, 10, 18, 36, and 37). In addition, 12 lead safety ECGs were obtained on Day 1 and pre-dose from Day 8 to 37 and 1, 2.5, 4, 6, 8, 12, and 16 h post-dose. 

Hourly mean HR data was determined over a 0–12 h interval on Days 1, 9, 10, 18, 36, and 37 to assess HR_AUEC_. The nadir of hourly mean HR was defined as the lowest whole-hour value measured during the 0–12 h interval.

### 4.4. Pharmacokinetic Assessments

Blood samples of about 3 mL were collected in ethylene di-amine tetra acetic acid (EDTA) tubes. 

For measurement of ponesimod plasma concentrations, blood was sampled pre-dose and at 1, 2.5, 4, 6, 8, 12, 16, and 24 h post-dose on Days 1, 18, and 37. Trough plasma concentrations (C_trough_) of ponesimod were measured on Days 19, 21, 23, 25, 27, 38, 39, 40, 41, and 42.

For cenerimod, C_trough_ was measured every second day on Day 10–36, each day on Day 37–43 as well as on Day 51, 65, 79, and 93. Cenerimod pharmacokinetic (PK) parameters were assessed on Days 9 and 36 with blood samples taken 1, 2.5, 4, 6, 8, 12, 16, and 24 h after cenerimod administration.

After centrifugation, plasma was transferred into a polypropylene tube and stored at ≤−70 °C (± 5 °C) pending analysis.

Plasma concentrations of cenerimod and ponesimod were determined using a validated liquid chromatography coupled to tandem mass spectrometry (LC-MS/MS) assay with a lower limit of quantification of 0.1 and 1 ng/mL, respectively, and the method was linear in the concentration range 0.1–100 and 1–1000 ng/mL, respectively [7,19]. Analysis of quality control samples of all runs showed that inter-batch coefficients of variation (precision) were <5.4% for ponesimod and <8.0% for cenerimod, whereas the average intra-batch accuracy was in the range 92.5–105.8% for ponesimod and 96.0–100.7% for cenerimod.

Non-compartmental PK analyses were performed using Professional WinNonlin 6.1 software (Pharsight Corp., Mountain View, CA, USA). The variables maximum plasma concentration (C_max_) and time to reach C_max_ (*t*_max_) were directly obtained from the plasma concentration–time profiles, area under the plasma drug concentration–time curve (AUC) from 0 to 24 h (AUC_0–24_) was calculated using the trapezoidal method [20], and *t*_1/2_ was calculated as ln2/λz, where λz is the terminal elimination rate constant estimated by log-linear regression analysis.

### 4.5. Lymphocyte Count Determination

Analysis of the lymphocyte count (pharmacodynamic (PD) biomarker) in peripheral blood was performed pre-dose on Days 1, 8, 9, 10, 13, 16, 18, 19, 22, 25, 28, 31, 34, 36, and 37. In addition, a 4 h post-dose assessment was performed on Days 1, 9, and 18. Measurements were also performed at each outpatient visit and EOS. Assessment of lymphocyte count was part of the clinical hematology evaluation. To assess the lymphocyte count, blood samples of 2.7 mL were collected into a K3-EDTA polypropylene tube and analysis was performed using a cell counter.

### 4.6. Safety Assessments

Safety and tolerability were assessed based on AEs including those of special interest (i.e., bradycardia (HR < 40 bpm), AV block, dyspnea, or elevated liver enzymes) as well as clinical laboratory and vital sign data (including BP). In addition, physical and neurological examinations were performed.

### 4.7. Statistical Analysis

Statistical analysis of HR_AUEC_ was performed using Student’s *t*-test for the comparison treatment versus placebo and Day 1 versus Day 9, 18, and 37. Differences were considered to be statistically significant at *p* < 0.05. The PK variables were analyzed descriptively providing the geometric mean and 95% confidence interval (CI) for C_max_, AUC_0__–24_, AUC from 0 to infinity (AUC_0__–∞_), and *t*_1/2_, and the median with the range for *t*_max_. The PD and cardiodynamic data are expressed as mean ± standard deviation (SD). Safety and tolerability data were analyzed descriptively by treatment group.

GraphPad Prism 7 (La Jolla, CA, USA) was used for the statistical analysis of the cardiodynamic data (HR_AUEC_) and descriptive statistics of clinical and PK data.

## Figures and Tables

**Table 1:** Pharmacokinetic parameters of ponesimod and cenerimod.

	Ponesimod					Cenerimod		
	Day 1	Day 18	Day 37	Day 18/Day 1	Day 37/Day 1	Day 9	Day 36	Day 36/Day 9
AUC_0__–∞_ (ng.h/mL)	NC	1997 (1673; 2384)	1748 (1481; 2063)	NC	NC	NC	14,634 (11,214; 19,097)	NC
AUC_0__–24_ (ng.h/mL)	864 (739; 1011)	838 (730; 962)	750 (658; 855)	0.97 (0.91; 1.04)	0.87 (0.81; 0.93)	140 (112; 176)	959 (751; 1225)	6.86 (6.35; 7.40)
C_max_ (ng/mL)	61.6 (50.8; 74.6)	56.5 (49.7; 64.2)	54.5 (48.4; 61.3)	0.92 (0.82; 1.03)	0.89 (0.79; 0.99)	9.2 (7.07; 12.0)	46.2 (36.5; 58.5)	5.02 (4.53; 5.56)
*t*_max_ (h)	3.25 (2.50; 4.00)	4.00 (2.50; 4.00)	2.50 (2.50; 4.00)	NC	NC	4.00 (4.00; 6.00)	4.00 (2.50; 6.00)	NC
*t*_1/2_ (h)	NC	29.0 (25.1; 33.5)	28.2 (24.7; 32.2)	NC	NC	NC	404 (340; 481)	NC

Treatment consisted of single-dose administration of 10 mg ponesimod alone (Day 1), at approximately 50% steady state of 2 mg once daily cenerimod (Day 18), and at steady-state cenerimod (Day 37) and multiple-dose administration of 2 mg cenerimod (from Day 9 to Day 36). Data are expressed as geometric mean (95% confidence interval (CI)) for AUC_0__–∞_, AUC_0__–24_, C_max_, and *t*_1/2_. Data for *t*_max_ are expressed as median (range). Geometric mean ratios are presented with 90% CI. AUC_0__–∞_ = area under the curve from zero to infinity; AUC_0__–24_ = area under the curve from 0 to 24 h; C_max_ = maximum concentration; NC = not calculated; *t*_1/2_ = apparent terminal half-life; *t*_max_ = time to C_max_.
